# The potential of eye-tracking as a sensitive measure of behavioural change in response to intervention

**DOI:** 10.1038/s41598-018-32444-9

**Published:** 2018-10-02

**Authors:** Sue Fletcher-Watson, Sarah Hampton

**Affiliations:** 1 Patrick Wild Centre, Division of Psychiatry, The University of Edinburgh, Edinburgh, UK; 2 Autism Research Centre, University of Cambridge, Cambridge, UK

## Abstract

One challenge to the development of effective interventions to support learning and behavioural change in neurodevelopmental disorders is a lack of suitable outcome measures. Eye-tracking has been used widely to chart cognitive development and clinically-relevant group differences in many populations. This proof-of-concept study investigates whether it also has the potential to act as a marker of treatment effects, by testing its sensitivity to differential change over a short period of exposure to an iPad app in typically developing children. The app targets a key skill in early social communication development, by rewarding attention to people, operationalised via a finger-tap on screen. We measured attention to images taken from the app, and a selection of matched stimuli to test generalisation of effects, at baseline and two weeks later. Children were assigned to either an app-exposure or no-app condition in the intervening period. The app exposure group showed increases in fixation on people for images from the app, and for distant-generalisation photographs, at high levels of complexity. We conclude that, with careful selection of stimuli, eye-tracking has the potential to make a valuable contribution to the range of outcome measures available for psycho-behavioural interventions in neurodevelopmental disorders.

## Introduction

The challenges of measuring outcome in intervention studies with populations with neurodevelopmental disorders have been well-documented^1–3^. Insensitive or inappropriate outcome measurement has been blamed for failures to demonstrate treatment effects in response to translational therapeutics^4^ and presents an obstacle to comparison across trials^5^. A good quality outcome measure for psycho-behavioural intervention should meet a series of criteria including: sensitivity to change; objective measurement; clinical relevance; construct validity; feasibility; and capable of being administered by assessors blind to status. Eye-tracking has been demonstrated as having potential to meet almost all of these criteria in experimental studies with populations with neurodevelopmental disorders. Below, we briefly review key evidence for these measurement properties and conclude that sensitivity to change – in particular, differential change between intervention and control groups – remains to be demonstrated.

Looking time to visual, or audio-visual, stimuli has long been used to represent cognitive constructs, such as memory, learning and attention. For example, the classic ‘preferential-looking’ paradigm uses the distribution of looking time between two stimuli on screen to infer cognitive processes^6,7^. In one example, by analysing looking time to pairs of stimuli distinguished either by (a) the number of circles represented or (b) the total surface area covered by the array, Libertus and colleagues demonstrate that numerical sense is represented by abstract number rather than the perceptual feature of cumulative surface area^8^. The use of an eye-tracker enhances this basic paradigm by permitting analysis not just of the overall distribution of looking time, but also the exact distribution and timing of eye-movements to specific regions within an image^9^.

Eye-tracking has proven particularly informative in two domains. First, eye-tracking has been used to provide insight into the cognitive processes of infants^10,11^. This literature has revealed the onset in the first few months of life of complex abilities such as relational memory^12^ and interpretation of intention^13^. Longitudinal work has related early cognitive abilities, probed through eye-tracking, with individual differences and diagnostic outcomes, resulting in tentative biomarker proposals for specific disorders^14,15^. Second, eye-tracking has been used to reveal subtle differences between groups including comparisons between clinical and typical populations across the life span. Group comparison studies have demonstrated attention patterns associated with affective disorders^16^, addiction^17^, and premature birth^18^ among others. In addition, eye-movement patterns have been related to clinical measures of symptom severity in some populations^19,20^. A recent addition to this literature explicitly demonstrates strong associations between eye-movement patterns and caregiver ratings on clinical measures of social communication, in a sample of children with autism^21^.

Thus eye-tracking has shown sensitivity to between-group differences, sensitivity to change over longer time spans, and some (albeit limited) clinical and construct validity – all features relevant to the evaluation of psycho-behavioural interventions. Moreover, it permits a blinded analysis of data, and provides a degree of objectivity. This is not absolute, due to the need to process raw data into meaningful variables for analysis, but may exceed that permitted by the use of self-report measures, or ratings of video behaviour - both commonly used in measurement of treatment effect for psycho-behavioural intervention. The use of eye-tracking in infant populations is widespread, and demonstrates how accessible this tool is to non-verbal populations and/or those with little or no conscious behavioural control. Thus, in relation to our original criteria, we can see that eye-tracking offers potential for objective measurement; clinical relevance; construct validity; feasibility; and capacity for blinded administration. However, differential sensitivity to change in the context of an intervention study has yet to be tested. In particular, it remains to be demonstrated whether a behavioural intervention in a real-world 3-d environment can influence the location of fixations or speed of fixation to those locations.

As a result of these strengths, eye-tracking has been incorporated into some pharmaceutical trial designs where eye-movements have occasionally been recorded as a measure of low-level oculomotor control^16,22^. In addition, some experimental studies report on eye-tracking used both as the measure of outcome and also as the training medium^23,24^. However the use of eye-tracking to capture fundamental, differential group change, reflective of high-level cognition, in response to psycho-behavioural intervention remains untested.

In the present study we aim to test the suitability of eye-movement recordings as a way to detect differential change over a short time span. Specifically, we ask whether eye-movement patterns can be used to distinguish between typically-developing children who have, and have not, been exposed to an iPad^TM^ app called *FindMe*. The app was originally designed for use by pre-school aged children on the autism spectrum^25^ and was trialled with that population^26^. The app reinforces, through the use of reward tokens and audio feedback, the behavioural response of tapping people on the screen. This behaviour was originally targeted as a key skill, with a foundational role in social communication development relevant to autism^27^. In this study however, our goal is not to capture a treatment effect, but instead to offer a proof-of-concept test that behavioural exposure can be reflected in eye-movements, with sufficient sensitivity to reveal differential change between groups. Thus, we report on data in a typically developing group of children. If we can demonstrate differential group change in eye-movement data, future studies will need to extend this work to a clinical population, including consideration of the design of eye-tracking tasks which have relevance to therapeutic goals.

In the current context, working with typically-developing toddlers, we hypothesise that repeated use of the app will (a) increase, or (b) speed-up visual fixation on people, because tapping this content is rewarded within the app. We do not expect any changes over time in looking to the whole scene. In other words, exposure to the app will specifically enhance attention to rewarded content.

It is possible that, as we are working with a non-clinical population, we will find that attention to people does not increase over time because it is already effectively at ceiling when baseline data are collected. We will attempt to probe this possibility by including stimuli with increasing quantities and variety of content (e.g. people, animals, toys) which can provide interesting targets for the gaze of the viewer. We expect that, if change over time is demonstrated in the app exposure group, the greatest effects will be seen in those stimuli at higher complexity levels, where there will be the biggest room for an increase (i.e. the lowest amount of fixation on people at baseline).

Psycho-behavioural intervention studies often ask whether treatment effects generalise to non-taught context. To address the issue of generalisation, we also ask whether any exposure-related changes in fixation patterns observed in the participants will also be replicated in fixation patterns to scenes with related but novel content. We predict that, if behavioural change is observed for scenes drawn from the iPad app being used for the exposure phase of the study, these changes will also be replicated for scenes with related but novel content. The degree of exposure-related effects may weaken for images which are more distantly related to the original stimulus set.

## Results

**Figure 1:**
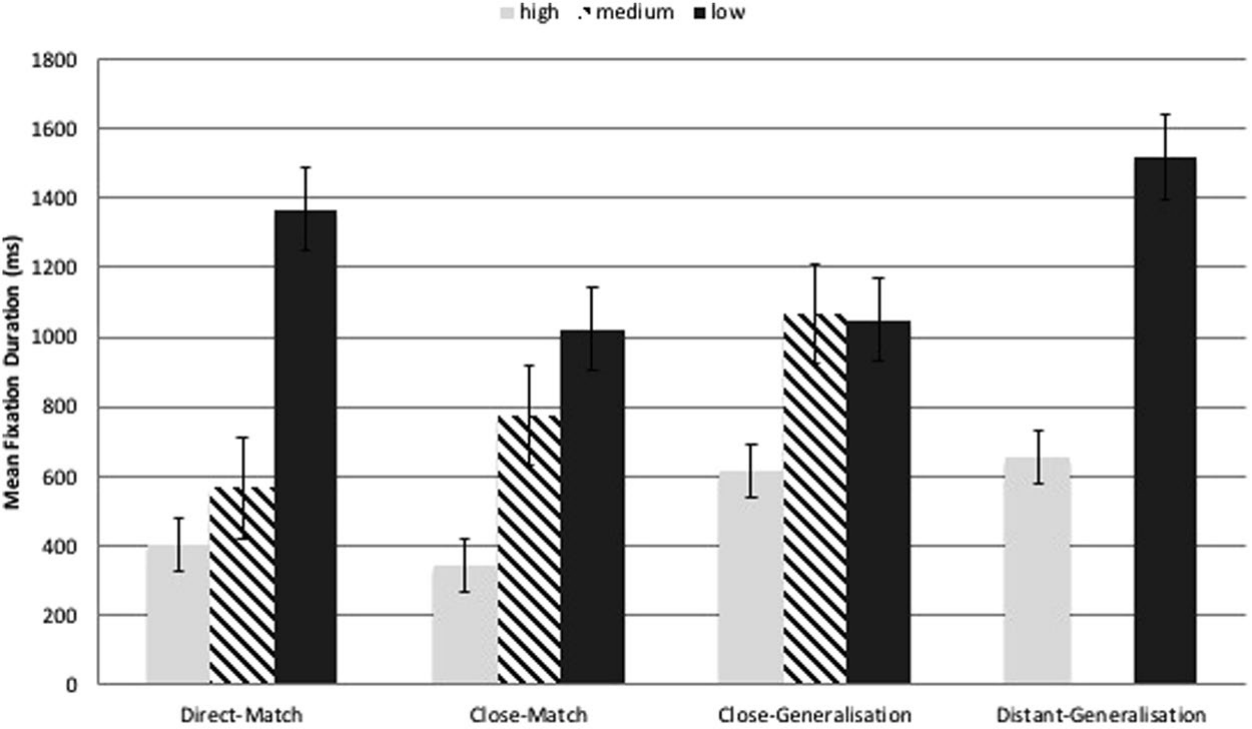
Mean baseline fixation duration by complexity level.

Looking times to people at baseline were smaller for stimuli at higher levels of complexity (see Fig. 1) indicating that, as predicted, there was more ‘room for improvement’ in looking at people for the ‘high complexity’ stimulus category. Paired t-tests by group on total fixation duration for the whole stimulus, for each stimulus category and complexity level separately, revealed no significant differences between Baseline and Outcome.

NB: There are no medium-complexity images in the distant-generalisation condition. See Methods for full details.

A repeated measures ANOVA on direct-match stimuli, people AoI (fixation duration change scores), contrasting complexity level (low, medium, high) and group (exposure vs control) revealed no main effect of group (p = 0.703) or complexity level (p = 0.093), nor any significant interaction (p = 0.110). Planned post-hoc tests showed that while there was no effect of group on change scores for low and medium complexity stimuli (both p > 0.3) there was a significant difference between groups in change in fixation duration to people for high complexity stimuli (t_(37)_ = 2.18, p = 0.035; mean difference = 258 ms; 95% CI: 18 ms–497 ms). This reflects increased looking time in the exposure group and an approximately stable looking time in the control group (see Table 1).

**Table 1:** Change Scores by Group for each Stimulus Type.

Complexity level:	Low		Medium		High	
Group:	Exposure	Control	Exposure	Control	Exposure	Control
**Fixation Duration** ***mean (SD)***						
Direct-Match *(FindMe iOS)*	−264 ms (699 ms)	−41 ms (947 ms)	−59 ms (260 ms)	−162 ms (345 ms)	215 ms (428 ms)	−42 ms (302 ms)
Close-Match (*FindMe Android*)	−225 ms (743 ms)	−147 ms (656 ms)	−112 ms (502 ms)	−116 ms (470 ms)	59 ms (305 ms)	−125 ms (397 ms)
Close-Generalisation *(Nosy Crow)*	−8 ms (373 ms)	25 ms (562 ms)	38 ms (542 ms)	4 ms (492 ms)	29 ms (456 ms)	−31 ms (460 ms)
Distant-Generalisation *(photographs)*	−19 ms (466 ms)	−253 ms (456 ms)	—	—	76 ms (392 ms)	−149 ms (285 ms)
**Time to First Fixate** ***mean (SD)***						
Direct-Match *(FindMe iOS)*	−136ms (503 ms)	11 ms (683 ms)	184 ms (733 ms)	12 ms (838 ms)	−184ms (689 ms)	−47ms (1122 ms)
Close-Match *(FindMe Android)*	75 ms (513 ms)	−262ms (723 ms)	−41 ms (829 ms)	−12 ms (791 ms)	−283 ms (801 ms)	113 ms (949 ms)
Close-Generalisation *(Nosy Crow)*	47 ms (522 ms)	133 ms (536 ms)	−53 ms (755 ms)	−49 ms (569 ms)	192 ms (757 ms)	249 ms (764 ms)
Distant-Generalisation *(photographs)*	−65 ms (772 ms)	191 ms (523 ms)	—	—	−229 ms (612 ms)	108 ms (645 ms)

*Significant group differences are marked in bold text.

The same analysis for close-match and close-generalisation stimuli revealed no significant effects in either ANOVA or planned comparison tests. However for the distant-generalisation stimuli there was a main effect of group (F (1, 37) = 4.72, p = 0.036) corresponding to a slight reduction in looking time to people by the control group compared to a plateau or increase in looking time to people in the exposure group. Planned comparisons showed that this group difference was non-significant for simple images but high complexity photos produced a statistically significant effect (t (37) = 2.06, p = 0.046; mean difference = 225 ms; 95% CI 4 ms–447 ms). The same analyses were performed looking at change scores in time to first fixate and revealed no effects of group, complexity level, nor any interactions.

## Discussion

This study asked whether recordings of eye-movement patterns show promise as a sensitive measure of behavioural change over time, whereby a change in eye-movement patterns can be detected as a result of rewarding a manual motor behaviour. Specifically, we probed whether the eye-movements of typically-developing children showed revealed differential change over time dependent on exposure to an iPad^TM^ app. We found that exposure to the app was associated with a specific and significant increase in looking time to rewarded content (i.e. people) which was not found in the control group. However, this pattern was only found for stimuli at high levels of complexity and for total looking times, not speed of fixation. This may be because looking times to people for low and medium complexity stimuli were already at ceiling, since these images did not incorporate a great deal of alternative content to attract fixation. The evidence of differential group changes over time was not replicated for close-match or close-generalisation stimuli that were drawn from similar, cartoon-style app screenshots. However, we did see a replication of the effect for photographs, again apparent in high-complexity images only.

A good outcome measure should be objective, sensitive to subtle changes over time, and able to detect clinically-meaningful group differences. Finding such measures which are applicable to psycho-behavioural interventions in children has proven challenging. These results provide very preliminary evidence that eye-tracking may offer a new way to capture treatment effects. First, eye-tracking is an objective measure, providing an opportunity to assess behavioural change in an assessment which can be blinded, and in any case is minimally susceptible to influence by either the administrator or others present (e.g. parents). In this regard, eye-tracking provides significant advantages over often-used directly observed measures to capture treatment effects, such as ratings of parent-child interactions. These data further indicate that, if stimuli are carefully selected to avoid ceiling effects, eye-tracking may capture behaviour changes even over very short periods of time – in this case just two weeks. Such ceiling effects might be less apparent in an application to a clinical population, when lower baseline looking levels may be expected for the chosen stimulus types. Moreover, the evidence that these changes may be duplicated in eye-movement patterns for a distantly-related stimulus set supports the interpretation that this measure is capturing real behavioural change, rather than simple adaptation. We did not find evidence of practice effects in our control group, meaning that the eye-movement data reported here are maximally sensitive to differential change between intervention and control groups.

While this study offers a proof-of-concept demonstration of the efficacy of eye-tracking as a sensitive measure of change, for the work to be of relevance it needs to be translated into studies with clinical populations. The absence of significant effects across all stimulus types here, indicates how important selection of task stimuli and constraints may be. This would be intensified in the context of a study with a clinical population, since the selection of stimuli and looking metrics of interest must also have relevance to the therapeutic goals of the intervention. Any such work should also be clear about the role of looking behaviour as an index of behavioural change. It is unlikely that looking behaviour would ever be considered an outcome in its own right, but with careful task design, it may serve as an index of behavioural change that represents a high-level cognitive shift, having meaning within the specific clinical and intervention context being studied.

Future investigations should build on this early work by investigating how eye-movement patterns may be linked to changes relevant to interpersonal interventions (e.g. parent training, therapist-delivered techniques) and their relation to clinical outcomes. In particular, extending this research to uncover whether eye-movements might change in response to exposure to an interpersonal experience, such as a therapist-delivered intervention, is crucial to determine the relevance of eye-movement measures for use in clinical trials.

## Method

### Design

The study used a two-group experimental design. All children were assessed at baseline by recording their eye-movements while freely viewing images drawn from the *FindMe* app and related content (see *Materials* for more detail). Children were then assigned to either an iPad exposure, or a control group. The Exposure group were given an iPad with the *FindMe* app to play at home over a two week period. At the end of this period, all children were invited back to the lab for an identical repeat of the baseline eye-tracking assessment.

### Participants

Typically-developing children aged 20–26 months were recruited through social media, parent and toddler groups, nurseries and a database of parents who had registered interest in taking part in research studies. Exclusion criteria were known neurodevelopmental difficulties and prior familiarity with the *FindMe* app. Ethical approval was given by the University of Edinburgh Psychology Research Ethics Committee and written informed consent was obtained from parents. The study followed ethical guidelines from the British Psychological Society.

The exposure group comprised 19 children (20–25 months) and the control group comprised 20 children (20–16 months, see details in Table 2). Three further participants were recruited and assigned to the control group but dropped out prior to their outcome appointment: their data are not included in subsequent analyses. There were no drop-outs from the exposure group. The groups were group-wise matched on child’s age, parental educational level, the number of days between baseline and outcome appointments, MacArthur-Bates Communicative Development Inventory: Words and Sentences (CDI) scores at baseline, child’s use of technology, child’s ethnicity and child’s number of siblings (see Table 2).

**Table 2:** Baseline characteristics for the intervention and control groups.

Variable	Exposure (n = 19)	Control (n = 20)	Statistic (Student’s t or X^2^)
Age (months) *mean (SD)*	23.06 (2.06)	23.25 (1.91)	t(37) = −0.30, p = 0.766
Length of exposure (days) *mean (SD)*	13.63 (1.30)	13.95 (2.84)	t(37) = −0.45, p = 0.658
MCDI^ words produced *mean (SD)*	173.2 (167.7)	187.6 (144.9)	t(37) = −0.29, p = 0.775
Hours TV / DVDs per week mean (SD)^*^	4.75 (6.33)	7.25 (4.06)	t(25.76) = −0.85, p = 0.402
Minutes gaming per week *mean (SD)*	31.68 (45.7)	54.25 (108.9)	t(37) = −0.61, p = 0.545
Gender *male: female*	11: 8	10: 10	X^2^ (1) = 0.244, p = 0.621
Ethnicity *white: other*	16: 3	17: 3	X^2^ (1) = 0.292, p = 0.589
Maternal education *Graduate: non-graduate*	16: 3	19: 1	X^2^ (1) = 1.232, p = 0.267
Siblings *Zero: one: two or more*	10: 4: 5	8: 11: 1	

*Equal variances not assumed.

^^^MacArthur-Bates Communicative Development Inventory.

### Materials and Apparatus

**Figure 2:**
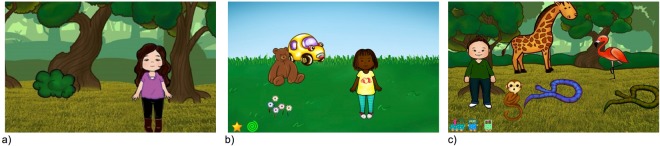
Direct-match images from the free-viewing task showing three complexity levels: (**a**) low; (**b**) medium; (**c**) high. (source: FindMe app).

The study employed a free-viewing task. The stimuli consisted of 48 images in total (see Fig. 2 for examples). *Direct-match* stimuli were 12 scenes from the *FindMe* app (iOS version), four each at three complexity levels (high, medium, low). App images of low complexity contained one person and no foreground objects. Images of medium complexity contained one person and up to three foreground objects, where a maximum of one of these could be an animal. Images of high complexity contained one person and four or more foreground objects, with no limit on the number of animals present.

*Close-match* stimuli were 12 images from an Android^TM^ version of the *FindMe* app. This app has closely-related content but images were created by a different artist and therefore the visual style is very different. *Close-generalisation* stimuli were 12 cartoon images taken from children’s apps created by the developer *Nosy Crow*^TM^. These images feature scenes from fairy tales. Both *close-match* and *close-generalisation* stimuli were sourced in low, medium and high complexity categories defined as for the *direct-match* stimuli. These images were chosen to be similar in form to those from the *FindMe* app: that is, images of one person facing forwards in an everyday scene.

Finally, *distant-generalisation* stimuli were 12 photographs sourced from Google^TM^ images. The photograph images were six photographs of high complexity and six photographs of low complexity. Low complexity photographs contained one person and no prominent foreground objects. High complexity photographs contained one person and at least one, prominent, foreground object. It was not possible to source photographs at three separate complexity levels due to lack of control over this content.

App images were presented in blocks according to level of complexity, with the low complexity images appearing first, followed by medium complexity images and finally high complexity images. Images appeared quasi-randomly according to stimulus category (FindMe iOS, FindMe Android, Nosy Crow) within each complexity level, with no more than two consecutive images belonging to the same stimulus category. Photographs were presented in a separate, final, block with images appearing randomly according to complexity level.

Each image was displayed for three seconds and followed by a one second display of a white fixation star in the centre of a black background. Between image blocks, ‘attention grabbers’ were shown to maintain focus on the screen. These were moving cartoon images of toys on a black background, accompanied by sound effects and displayed for three seconds (after every fourth image) or six seconds (between the high complexity block and photos block). Total eye-tracking time was approximately 4 minutes, presented in one sequence.

Images were all 1000 × 810 pixels. Eye movements were recorded using a Tobii© x60 eye-tracker. Stimuli were presented and eye movements recorded using Tobii Studio 3.1.0 software. Images were presented on an HP EliteDesk 800 G1 SFF with screen size width 51.0 cm and height 28.5 cm. The Tobii x60 system tracks both eyes to a rated accuracy of 0.3 degrees, sampled at 60 Hz.

### Procedure

At baseline, children sat on their parent’s lap approximately 50–60 cm from the monitor and viewed the images while their eye-movements were recorded. Eye-tracking calibration was performed using a five-point system and inspected by the researcher. Parents were instructed not to distract their child during the task and not to direct their child’s attention to any particular aspect of an image.

During the baseline assessment, parents or guardians completed a background questionnaire regarding: parental education and profession, the child’s age, gender, ethnic background, number of siblings, history of developmental difficulties, familiarity with the *FindMe* app and use of technology. Parents also completed the MCDI, to confirm the language level of the children.

Following collection of baseline data, children were assigned (alternating assignment) to either exposure or control conditions. Those in the exposure group were given an iPad with the *FindMe* iOS app installed to take home and play, in between the baseline and outcome assessments. Parents were instructed to aim for their child to play the app for around 5 minutes a day, or 10 minutes every other day. Parents were asked to not interfere with their child’s playing of the app and to ensure that no-one other than their child played the app. Parents were given a diary to take home in which to write any comments they might have about their child’s use of the app.

All participants returned for the outcome assessment approximately two weeks after baseline (mean = 13.8 days; range = 9–21 days). During this assessment, participants completed the same free viewing task as at baseline, with the same stimuli. Additionally, parents in the exposure group were asked to estimate the frequency and duration of their child’s use of the app (mean = 80 minutes, SD = 75 minutes). This mean corresponds very closely to the requested play time of 5 minutes per day (equivalent to 70 minutes in total). Data was recorded by the *FindMe* app concerning the highest level reached (all children reached the maximum level of four), and the number of scenes completed (mean = 197 scenes; SD = 127 scenes).

### About the FindMe App

FindMe was originally created by researchers at the University of Edinburgh as a social skill development game for children with autism^24^. The app consists of cartoon scenes in which a character appears on screen in a simple, outdoor scene. The child must touch the character in order to receive a reward in the form of a small token in the corner of the screen (bottom, left hand side). Collection of five tokens in the app triggers a short animation (either spinning shapes with music, jumping acrobats with cheering, or a moving train with sound effects) which acts as a further reward for the player. As a child plays the game, the scenes become more complex with an increasing number of distracting objects such as toys or animals being presented. Recorded voice prompts serve to remind the child to respond and give clues as to how to play (e.g. *“Can you find me?”*).

### Analysis Methods

Analysis focused on mean fixation duration (mean FD), and time to first fixate (TTFF) for a single area of interest (AoI) which was the person. First, data were ‘cleaned’ by removing any trials in which total fixation duration to the whole scene, for the entire viewing period, was less than 500 ms. This resulted in exclusion of, on average, 12% of individual trials (see Table 3 for details). The distribution of missing data was such that there were no missing data at the level of mean scores for every participant, in every stimulus category. Second, change-scores were calculated. For mean FD, Baseline were subtracted from Outcome data to give a variable where a positive value indicated an increase in looking to a specific region over time. For TTFF, the calculation was reversed (Baseline minus Outcome). Thus, positive values can always be interpreted as an increase in attention or interest in the relevant region. Change scores not only capture the effects of exposure over time but also often produce a normal distribution permitting parametric analysis, which was the case here.

**Table 3:** Number of trials excluded due to inadequate data.

	Baseline			Outcome		
	Complexity level			Complexity Level		
	High	Medium	Low	High	Medium	Low
Direct-Match *(FindMe iOS)*	27 (16%)	28 (17%)	18 (11%)	24 (15%)	13 (8%)	11 (7%)
Close-Match *(FindMe Android)*	20 (12%)	30 (18%)	20 (12%)	18 (12%)	15 (10%)	12 (8%)
Close-Generalisation *(Nosy Crow)*	22 (13%)	29 (17%)	17 (10%)	28 (18%)	15 (10%)	9 (6%)
Distant-Generalisation *(photographs)*	33 (13%)	—	32 (13%)	18 (8%)	—	24 (10%)

NB: Baseline percentages are calculated from a total of n = 42 participants viewing 4 stimuli per category × complexity level or, in the case of distant-generalisation only, 6 stimuli per complexity level. Outcome percentages are calculated from a total of n = 39 participants viewing 4 stimuli per category × complexity level or, in the case of distant-generalisation only, 6 stimuli per complexity level. Inadequate data is defined as <500 ms total fixation duration to the whole scene.

Analyses use ANOVA to explore the between-subjects effects of app exposure vs control on mean FD and TTFF change scores, at three levels of stimulus complexity (low, medium, high) for each stimulus type separately (direct-match, close-match, close-generalisation, distant-generalisation). Between-subjects effects are explored for close-match stimuli first and then we seek evidence of a similar pattern for the other stimulus categories. In addition, planned comparisons use t-tests to probe group differences at each complexity level separately. Throughout, an alpha level of 0.05 is used for significance.

## Data Availability

Full data are available in an anonymised form at www.dart.ed.ac.uk/library Please search for “eye-tracking outcome” to locate the relevant library item.
